# Supplementary material for: Antimicrobial Stewardship Programmes: Healthcare Providers’ Perspectives on Adopted Hospital Policies That Combat Antibacterial Resistance in Selected Health Facilities in Uganda
Source: Antibiotics (Basel). 2024 Oct 23;13(11):999. doi: 10.3390/antibiotics13110999 (PMC11590866; doi:10.3390/antibiotics13110999)
Supplement: Supplementary file 1 [file antibiotics-13-00999-s001.zip › File S2. Questionnaire on antimicrobial stewardship.pdf]

Antimicrobial stewardship programmes: Healthcare providers' perspectives on adopted hospital policies to combat antibacterial resistance in selected health facilities in Uganda

Questionnaire number (*pre-fill*) ……………………………………………………………………

Date of the survey	
Data collector name (pre-fill)	

Section A: Please complete the information about the health facility

**1.**	Health facility I.D. (*pre-fill*)	
**2.**	City /town (*pre-fill)*	
**3.**	District *(pre-fill)*	
**4.**	Region *(pre-fill)*	
**5.**	Type of Health facility (*Tick one only)*	1. Regional referral hospital<br>2. General hospital<br>3. Private Not-for-profit hospital
**6.**	Nature of Health facility *(Tick one only)*	**1.** Teaching hospital.<br>2. Non-teaching hospital
**7.**	Number of licensed beds (pre-fill) **……………………..**	

B) **Demographics and general information**

1) Age ……………………… (Completed *in years*)

2) Sex: 1. Male ☐ 2. Female ☐

3) Current position in this facility ____________________

4) Department ______________________________

5) Level of training: (*Tick one only)*
1. Certificate ☐ 2. Diploma ☐ 3. Degree ☐ 4. Masters ☐ 5. Ph. D ☐

6) Type of health professional cadre: (*Tick one only)*

1.Nurse ☐ 2. Pharmacy Technician ☐ 3. Clinical officer ☐ 4. Medical doctor 5. ☐ Pharmacist 6. ☐ Medical Specialist, 7. ☐ Consultant

7) Duration of practice since completion of medical School………………………… (Completed *in years*)

C) **Attitude towards antimicrobial stewardship used to optimise antibacterial use and reduce the risk of antibacterial resistance development in this hospital**

b) The following statement(s) relate(s) with your knowledge and perceptions on antimicrobial stewardship

	**Strongly Disagree** (1)	**Disagree Slightly (2)**	**Neither agree nor disagree** (3)	**Agree** (4)	**Strongly Agree** (5)
1. I know what antimicrobial Stewardship is					
2. I am familiar with the goals of antimicrobial Stewardship programmes in this hospital					
3. Implementation of antimicrobial Stewardship programmes are essential in this hospital					
4. Antimicrobial stewardship involves the appropriate selection of antibacterials in this hospital					
5. Antimicrobial stewardship involves optimal administration of all antibacterials in appropriate doses, route of administration and duration of antimicrobial therapy in this hospital					
6. Antimicrobial stewardship interventions can improve patient outcomes if the selection of antibacterial doses, formulations					

and frequency of administration is well optimised in this hospital					
7. If well implemented, antimicrobial stewardship Strategies or interventions can reduce antimicrobial resistance in this hospital.					
8. Implementing antimicrobial stewardship practices can reduce the length of hospital stay of patients					
9. Implementing antimicrobial stewardship practices can increase appropriate antibacterial use in this hospital					
10. Antimicrobial stewardship strategies can decrease the incidence of C. *difficile* rates if well implemented.					
11. This hospital provides adequate training on antimicrobial stewardship practices					
12. Additional staff education on antimicrobial stewardship practices is needed.					

D) **Antimicrobial stewardship practices used by healthcare providers to optimise antibacterial use and reduce the development of resistance**

1) Healthcare providers use local guidelines to initiate prompt, effective antibacterial treatment within one hour of presentation or as soon as possible in patients with life-threatening infections

1. Yes 2. No 9. I don't know

2) Healthcare providers avoid unnecessary use of broad-spectrum antibacterial agents.

1. Yes 2. No 9. I don't know

3) Healthcare providers document in the prescription chart and patients' clinical records the clinical indication, route, dose, duration and review date of antimicrobials
   1. Yes 2. No 9. I don't know

4) Healthcare providers use only single doses of antibacterials for surgical and other procedures for which prophylaxis is effective unless the duration of the operation/procedure is prolonged, there has been excessive blood loss or published national recommendations suggest otherwise
   1. Yes, 2. No 9. I don't know

5) Healthcare providers switch to the correct antibacterial agents when susceptibility testing indicates resistance or to a cheaper or more cost-effective antibacterial that is also compatible with the clinical presentation
   1. Yes, 2. No 9. I don't know

6) Healthcare providers review antimicrobial prescriptions for hospital inpatients on all ward rounds
   1. Yes 2. No 9. I don't know

7) Healthcare providers appropriately choose one of the five antibacterial prescribing decisions 48 hours after initiating antimicrobial treatment such as Stop antibacterials if there is no evidence of infection, switch antibacterials from intravenous to oral administration, change antibacterials – ideally to a narrower spectrum or broader if required, continue and review again at 72 hours.
   1. Yes 2. No 9. I don't know

8) Healthcare providers educate patients, caretakers, nurses, or other supporting clinical staff about when antibacterials are not required or administer antibacterial agents in compliance with the dose, duration, and frequency of administration of their prescribed antimicrobial.
   1. Yes, 2. No 9. I don't know

9) Healthcare providers engage the views of others involved in antimicrobial treatment policy decisions, including championing best practice, and that care team has to co-operate with others more expert than oneself when such expertise is required.
   1. Yes 2. No 9. I don't know

10) Healthcare providers have regular engagement in the team-based measurement of the quality and quantity of antibacterial use and understanding that this should be shared with prescribers, as well as informing antimicrobial surveillance/infection prevention and control measures
   1. Yes, 2. No 9. I don't know

11) Healthcare providers use locally agreed process measures of quality (e.g. compliance with guidance), outcome and balancing measures, such as unintended adverse events or complications.

1. Yes, 2. No 9. I don't know

12) Using the results of adverse event monitoring, laboratory susceptibility reports, antimicrobial prescribing audits, and antimicrobial usage data to inform, promptly, best antimicrobial prescribing practices produce sustained improvements in patient care quality.

1. Yes 2. No 3. I don't know

E) **Perceived level of antibacterial resistance and its impact on optimisation of antibacterial use in the management of childhood infections in health facilities in Uganda**

To what extent do you think antibacterial resistance is affecting the country, the hospital or ward (*Rank them on the scale of 1-5 (where 0 is I do not know; "1" is not important; "2" is less important; "3" is important and "4' very important)*

<u>Tick or choose one only below</u>

Perceived level of antibacterial resistance	I don't know (0)	Not important (1)	Less important (2)	Important (3)	Very important (4)
1) At what scale would you put the level of antibacterial resistance in this **country**?					
2) At what scale would you put for the level of antibacterial resistance in this **hospital**?					
3) At what scale would you put the level of antibacterial resistance on your **ward or unit**?					
4) The level of antibacterial resistance influences the choices of antibacterial prescriptions?					
5) What is the scale of antibacterial-resistant infections in children under five in this hospital?					
6) What is the scale of antibacterial resistance among first-line antibacterial agents for common bacterial infections in children under-five?					
7) How do you rate the impact of antibacterial resistance on patient clinical outcomes and safety in your department or ward or unit?					

F) **Healthcare providers' ranking of importance possible causes of antibacterial resistance in this health facility**

What are some of the factors contributing to antibacterial resistance in your health facility? (*Rank them on the scale of 1-4 (where "1" is not important; "2" is less important; "3" is important and "4" very important)*

<u>Tick or choose one only below</u>

Cause of antibacterial resistance	Not important (1)	Less important (2)	Important (3)	Very important (4)
1. Prescribing the wrong antibacterial drugs				
2. Prescribing antibacterials when not needed				
3. Poor adherence of patients to prescribed antibacterial courses				
4. Poor access to treatment guidelines within the hospital				
5. Lack of continuing education and updated information on antibacterials for prescribers				
6. Empirical (without lab investigation) antibacterial prescribing				
7. Poor access to antibiograms to guide prescription				
8. Use of antibacterials for a longer duration than the standard duration				
9. Lack of sufficient diagnostic laboratory facilities				
10. Lack of/inadequate infection control in the health facility				
11. Lack of restriction controls on antibacterials access and prescription				
12. Lack/shortage of antibacterials				
13. Poor quality antibacterials				
14. Pharmaceutical company influence				
15. Others (specify -- -- -- -- --				

G) **Perspectives of healthcare providers on adopted hospital policies to combat antibacterial resistance in health facilities in Uganda**

1) Has the hospital strengthened regulations and implemented the national policy on providing and distributing high-quality antibacterials?
   1. Yes 2. No 9. I don't know

2) Has the hospital developed and disseminated standard treatment guidelines and protocols for prophylaxis of infectious diseases?

   1. Yes 2. No 9. I don't know

3) Has the hospital participated in a nationwide or regional antibacterial awareness campaign targeting both public and private health facilities, including targeting specific groups (e.g. doctors, pharmacists, nurses, and hospital administrators) in Uganda's public and private health facilities?
   1. Yes 2. No 9. I do not know

4) Does the hospital regularly review antibacterials from the national essential medicines lists (EML) and adjust with the hospital formulary?

   1. Yes 2. No 9. I don't know

5) Has the hospital collaborated and participated in translating international and national action plans into hospital action plans to support antimicrobial stewardship programmes and fight antibacterial resistance?

   1. Yes 2. No 9. I don't know

6) Has the hospital implemented a Medicine Therapeutic Committee (MTC) and AMS subcommittee to regulate, monitor and optimise antibacterial use?

   1. Yes 2. No 9. I don't know

7) Has the hospital monitored antibacterial consumption, identified areas for improvement, and designed a system for surveillance of antibacterial use to match monitoring the national level of antibacterial consumption at health facilities?
   1. Yes 2. No 9. I don't know

8) Has the hospital generated reports on antibacterial resistance and used them to guide the prescription and use of antibacterials?
   1. Yes 2. No 9. I don't know

9) Is there a functioning hospital antimicrobial resistance surveillance system covering antibacterials in hospitals and outpatient clinics, with external quality assurance and a national coordinating centre producing reports on resistance levels?

1. Yes 2. No 9. I don't know

10) Is there a hospital infection prevention and control Committee (IPC) with an available operational plan or standard operating procedures (SOPs)?

1. Yes 2. No 9. I don't know

H) **Antimicrobial stewardship (AMS) programmes and AMS techniques used to optimise antibacterial use and minimise antibacterial resistance**

a) Is there any existing formal antimicrobial stewardship program in this hospital?
Yes 2. No 9 I don't know

Which of these antimicrobial stewardship strategies/interventions is/are used in this hospital to optimise antibacterial use or reduce resistance development? (*check all that apply*)

1) Broad/key interventions to optimise antibacterial use and reduce antibacterial resistance in this hospital

	Compulsory in all areas (3)	Voluntary /Optional in some areas (2)	Not feasible (not applicable) (1)	Don't know (0)
**Policy**				
**1.** Does your ward, unit or hospital have a policy that requires prescribers to document in the medical record or during order entry the dose, duration, and indication for all antibacterial prescriptions?				
**2.** Is there a policy requiring the ward, unit or hospital healthcare team to review the appropriateness of all antibacterials prescribed 48 to 72 hours after the initial order (e.g. "**antibacterial time out**")?				
**Broad interventions to improve antibacterial use**				
**1.** Do specified antibacterial agents need to be approved by the head or senior clinician of the unit before prescribing, dispensing, or administration (i.e. **pre-authorisation**)?				

**2.** Does the head of ward or unit or senior clinician review treatments given to patients using specified antibacterial agents and provide feedback (e.g. **prospective audit with feedback**)?				
**3.** Does the hospital have Antimicrobial formulary restriction and approval systems?				

2) ***Specific strategies implemented in hospital or ward or unit to optimise antibacterial use and reduce the development of antibacterial resistance in children under-five with bacterial infections***

	Compulsory in all areas	Optional in some areas	Not feasible (not applicable)	Do not know
1. Does your hospital utilise antimicrobial order forms (requiring clinicians to justify their antibacterial use in a given patient or infections?				
2. Does your hospital utilise antimicrobial combination therapy (use of multiple antimicrobials) for initial empirical treatment of infections to increase the breadth of coverage?				
3. Does this hospital utilise **streamlining** (switching to a more targeted narrows spectrum antibacterial once an organism is identified via culture) or de-escalation (discontinuing the empirical antimicrobial if the culture is negative				
4. Does this hospital or ward utilise **dose optimisation** (i.e., account for individual patient characteristics such as age, renal function, and weight; causative organism; site of infection; and pharmacodynamics of the drug) when prescribing antimicrobials				
5. Does this hospital or ward or unit utilise a systematic plan for converting parenteral to oral (I.V. to P.O.)				

administration of antibacterials once a patient meets defined clinical criteria?				
6. Does the hospital or ward, or unit use standard treatment guidelines and clinical pathways to recommend the selection of appropriate antibacterial formulation, dose, and duration				
7. Does this hospital or ward, or unit use education techniques to educate prescribers about the appropriate prescription of antibacterials				
8. Are there diagnostic pathways for patients with reported bacterial infections or allergies?				
9. Are antibiograms on various organisms developed and distributed least annually and assessing antimicrobial resistance trends within the facility?				
10. Do prescribers use rapid diagnostic tests with/without stewardship advice?				
11. Does the hospital monitor hospital-specific antimicrobial resistance?				
12. Does the microbiology lab present selective reporting of susceptibility testing?				
13. Are prescribers educated on good antimicrobial prescribing practice and resistance?				
14. Has the hospital developed Clinical decision-support systems?				

l) **Which of the following barriers to antimicrobial stewardship have you encountered at your facility?** ***(Select all that apply)***

The barrier to implementing Antimicrobial stewardship	response	
1) Lack of training and education in antimicrobial use	1. Yes	2. No
2) Lack of time among the antimicrobial stewardship team	1. Yes	2. No
3) Lack of dedicated funding for an antimicrobial stewardship team	1. Yes	2. No
4) Lack of leadership to promote antimicrobial stewardship at the facility	1. Yes	2. No
5) Lack of support from senior clinicians at the facility	1. Yes	2. No
6) Lack of infectious diseases or microbiology services	1. Yes	2. No
7) Lack of pharmacy resources	1. Yes	2. No
8) Lack of willingness from healthcare providers to change their prescribing practices	1. Yes	2. No
9) Lack of enforcement by facility management/executive	1. Yes	2. No
10) Lack of an electronic medication management system	1. Yes	2. No
11) High level of transient or part-time staff	1. Yes	2. No
12) Inadequate time for Antimicrobial stewardship activities amongst healthcare providers	1. Yes	2. No
13) Personnel shortages	1. Yes	2. No
14) Inadequate funding for antimicrobial stewardship strategies, activities or personnel	1. Yes	2. No
15) Lower priority antimicrobial stewardship than other clinical initiatives	1. Yes	2. No
16) Inadequate information technology (I.T.) support antimicrobial stewardship practices	1. Yes	2. No
17) Opposition antimicrobial stewardship from healthcare providers	1. Yes	2. No
18) A paucity of data on improved outcomes with ASPs in Uganda	1. Yes	2. No

19) Financial support for ASP activities from government or other partners	1. Yes 2. No

Thank you so much for taking the time to respond to this questionnaire.
