# Supplementary material for: Antimicrobial Stewardship Programmes: Healthcare Providers’ Perspectives on Adopted Hospital Policies That Combat Antibacterial Resistance in Selected Health Facilities in Uganda
Source: Antibiotics (Basel). 2024 Oct 23;13(11):999. doi: 10.3390/antibiotics13110999 (PMC11590866; doi:10.3390/antibiotics13110999)
Supplement: Supplementary file 1 [file antibiotics-13-00999-s001.zip › File S3. Supplementary data for the manuscript.pdf]

Antimicrobial stewardship programmes: Healthcare providers' perspectives on the adopted hospital policies to combat antibacterial resistance

Table S1 Sociodemographic characteristics of respondents (N=582)

	Frequency	Percentage (%)
**Sex**		
Male	333	57.2
Female	249	42.8
**Age categories**		
30-39	246	42.3
20-29	96	16.5
40-49	171	29.4
50+	69	11.9
**Hospital department**		
Medicine	98	16.8
Surgery	71	12.2
Paediatrics	109	18.7
Pharmacy	50	8.6
Obstetrics and Gynaecology	93	16
Outpatient Department	101	17.4
Other department	60	10.3
**Type of professional cadre**		
Nurse	199	34.2
Pharmacy Technician	30	5.2
Clinical Officer	136	23.4

Medical doctor	121	20.8
Pharmacist	24	4.1
Medical specialist	50	8.6
Lab Technician	22	3.8
Total	582	100
**Level of academic training)**		
Diploma	327	56.2
Degree	191	32.8
Masters and above	64	11
**years_at_work**		
Less than 5 years	184	31.6
5<10	140	24.1
10 and above	258	44.3
**Region of Uganda**		
Central	193	33.2
North	67	11.5
East	174	29.9
West	148	25.4
**Type of health facility**		
Regional referral hospital	130	22.3
General hospital	396	68
Private-not-for profit	56	9.6
**Nature of the health facility**		
Teaching hospital	186	32
Non-teaching hospital	396	68
**Bed capacity**	N.	col%
100 beds	396	68
101- 300	37	6.4
Over 300	149	25.6

Table S2 showing the age distribution of study respondents

Age (years)	N	sum	mean	sd	p50	p25	p75
	582	22242	38.2	8.4	38	31	43

Table S3 age distribution of study respondents by sex

Age		
**Male**	mean	37.0
	standard deviation	8.3
	median	36
	Lower quartile	30
	Upper quartile	42
	minimum	23
	Maximum	60
**Female**	mean	39.8
	standard deviation	8.2
	median	39
	Lower quartile	33
	Upper quartile	43
	minimum	20
	Maximum	60
**Total**	mean	38.2

	standard deviation	8.4
	median	38
	Lower quartile	31
	Upper quartile	43
	minimum	20
	Maximum	60

alpha cronbach testing for reliability of the questionnaire

Perception of the burden of ABR

Test scale = mean(unstandardized items)

. alpha b1 b2 b3 b4 b5 b6 b7

Average interitem covariance:	0.4339467
Number of items in the scale:	7
Scale reliability coefficient:	0.8107

Perception on possible causes of ABR

Test scale = mean(unstandardized items)

alpha c1 c2 c3 c4 c5 c6 c7 c8 c9 c10 c11 c12 c13 c14

Average interitem covariance:	0.2423754
Number of items in the scale:	14
Scale reliability coefficient:	0.8459

Adoption of hospital policies to support antimicrobial stewardship programmes	
Test scale = mean(unstandardized items)	
alpha hb1 hb2 hb3 hb4 hb5 hb6 hb7 hb8 hb9 hb10 hb11 hb12	
Average interitem covariance:	0.7935159
Number of items in the scale:	12
Scale reliability coefficient:	0.9268

Proportionate number to size

We conducted the proportionate number to size allocation

Step 1. We determined the total number of healthcare providers to be allocated as 768 healthcare providers

Step2. We identified the groups to receive the allocation

1. Nurses 2. Pharmacy technicians 3. Clinical officers 4. Medical officers 5. Pharmacists 6. Medical specialists 7. Laboratory technicians

Step 3. We determined the size of the group (group population size)

Nurses =224, Clinical officers= 192, Pharmacy technicians =32, Medical officers =194, Pharmacists =32, Medical specialists =64, laboratory technicians =32

Step 4 : We calculated the proportion of each group relative to the total size

Nurses = 224/768, clinical officers =192/768, pharmacy technicians =32/768, medical officers =194/768

Step 5: We multiplied the proportion of each group with number of healthcare professional (like nurses) we found in each hospital studied to determine the total number of health professionals to be interviewed (Assuming hospital x has 80 nurses

Nurses = 224/768 x 80

Step 6: we rounded off numbers to get real number of number of health professionals in a group to interview

We selected healthcare providers using a proportionate number to size. We targeted 224 nurses, 192 clinical officers, 32 pharmacy technicians, 194 medical officers, 32 pharmacists, 64 medical experts, and 32 laboratory technicians out of the needed 768 healthcare providers. We computed the number of different professionals to be selected from each facility by dividing the number of people in a specific profession by the total number of health professionals to obtain the fraction of that profession at the facility. This fraction was then multiplied by the total number of health professionals to be sampled from the health facility. The different numbers of healthcare providers per health facility selected in the study are shown

	**N=582**	**Nurse =199**	**PT=30**	**CO=136**	**MO=121**	**P=24**	**MS=50**	**LT=22**	**M(1QR)**	**P value**
Scale of ABR in this ..country	537(92.2%)	171(85.9%)	27(90%)	126(92.7%)	118(97.5%)	24(100%)	49(98%)	22(100%)	4(3-4)	0.023
Scale of ABR in this ..hospital.	504(86.6%)	163(81.9%)	24(80%)	117(86%)	106(87.6%)	24(100%)	49(98%)	21(95.4%)	3(3-4)	0.212
Scale of ABR on your ward	464(79.7%)	151(75.9%)	25(83.3%)	107(78.9%)	97(80.1%)	23(95.8%)	45(90%)	16(72.8%)	3(3-4)	0.837

The level of ABR influences choices of antibacterial prescribed .	529(90.9%)	171(86%)	25(83.3%)	130(95.5%)	114(94.2%)	20(83.3%)	49(98%)	20(90.9%)	4(3-4)	0.025
What is the scale of resistant child hood bacterial infection	361(62.0%)	122(61.3%)	19(63.3%)	82(60.3%)	77(63.6%)	15(62.5%)	32(64%)	14(63.7%)	3(2-3)	0.943
What is scale of ABR among first line antibacterial i...	385(66.2%)	130(65.4%)	22(73.3%)	92(67.6%)	74(61.2%)	15(62.5%)	38(76%)	14(63.7%)	3(2-4)	0.289
How do you rate the impact of ABR on patient clinical outcomes ...	494(84.9%)	159(79.9%)	27(90%)	114(83.8%)	102(84.3%)	22(91.7%)	49(98%)	21(95.4%)	3(3-4)	0.019

Perception of causes of ABR among healthcare professionals

	**Possible causes of ABR**	N=582)	**Nurse =199**	**PT=30**	**CO=136**	**MO=121**	**P=24**	**MS=50**	**LT=22**	**M(1QR)**	**Pvalue**
1	Prescribing the wrong antibacterial drugs	486(83.5)	162(81.4)	23(76.7)	114(83.8)	99(81.8)	23(95.8)	44(88.0)	21(95.5)	4(3-4)	0.694
2	Prescribing an antibacterial when not needed	518(89.0)	177(88.9)	27(90.0)	119(87.5)	104(86.0)	23(95.8)	50(100.0)	18(81.8)	4(3-4)	0.148
3	Poor adherence of patients to prescribed antibiotic courses	524(90.0)	176(88.4)	28(93.3)	123(90.4)	107(88.4)	24(100.0)	49(98.0)	17(77.3)	4(3-4)	0.125
4	Poor access to treatment guidelines within hospital	376(64.6)	133(66.8)	15(50.0)	83(61.0)	80(66.1)	12(50.0)	38(76.0)	15(68.2)	3(2-4)	0.255

5	Lack of continuing education and updated information on antibiotics for pr...	469(80.6)	161(80.9)	24(80.0)	101(74.3)	97(80.2)	18(75.0)	49(98)	19(86.4)	3(3-4)	0.523
6	Empirical (without lab investigation) antibiotic prescribing	481(82.6)	164(82.4)	25(83.3)	105(77.2)	103(85.1)	22(91.7)	42(84.0)	20(90.9)	4(3-4)	0.281
7	Poor access to antibiograms to guide prescription	473(81.3)	156(78.4)	23(76.7)	106(77.9)	99(81.8)	21(87.5)	49(98.0)	19(86.4)	3(3-4)	0.008
8	Use of antibacterials for longer duration than standard duration	439(75.4)	170(85.4)	21(70.0)	96(70.0)	85(70.2)	11(45.8)	38(76.0)	18(81.8)	3(3-4)	0.001
9	Lack of sufficient diagnostic laboratory facilities	455(78.2)	151(75.9)	26(86.7)	104(76.5)	96(79.3)	19(79.2)	43(86.0)	16(72.7)	3(3-4)	0.91
10	Lack/inadequate infection control in the health facility	398(68.4)	137(68.8)	20(66.7)	87(64.0)	80(66.1)	14(58.3)	40(80.0)	20(90.9)	3(2-4)	0.077
11	Lack of restriction controls on antibacterials access and prescription	459(78.9)	155(77.9)	22(73.3)	104(76.5)	92(76.0)	22(91.7)	48(96.0)	16(72.7)	3(3-4)	0.041
12	Lack/shortage of antibacterials	365(62.7)	132(66.3)	21(70.0)	87(64.0)	70(57.9)	12(50.0)	35(70.0)	8(36.4)	3(2-4)	0.049
13	Poor quality antibacterials	384(66.0)	122(61.3)	20(66.7)	98(72.1)	76(62.8)	12(50.0)	43(86.0)	13(59.1)	3(2-4)	0.106
14	Pharmaceutical company influence	364(63.4)	121(60.8)	21(70)	94(69.1)	74(61.2)	13(54.2)	37(74.0)	9(40.9)	3(2-4)	0.283

Computation of relative importance index for perception on causes of antibacterial resistance among healthcare professionals

The relative Importance Indexx(RII) is a statistical measure used to evaluate the relative importance of various factors like causes of antibacterial resistance in a given population. RII calculates the importance of each factor based on frequency (how often is the

factor mentioned). In our study the healthcare provider would respond on the importance of the factor as a cause of antibacterial resistance. There was relative ranking of the factor by the respondent as well.

The RII score ranges from 0 (least important) to 1 (most important). A higher RII score indicates greater importance.

RII is calculated using the following formula

RII= summation (weighted scores) / maximum possible score

Where

Weighted scores = (frequency x ranking)

Maximum possible score = (number of Respondents x maximum Ranking

Relative index analysis was selected in this study to rank the criteria according to their relative importance. The following formula is used to determine the relative index RI =   w A × N (1) where w is the weighting as assigned by each respondent on a scale of one to five with one implying the least and five the highest. A is the highest weight and N is the total number of the sample. Based on the ranking (R) of relative indices (RI), the weighted average for the two groups will be determined.

Relative importance index is the sum of all the responses divide by the number of responses and the maximum number of likert scale i.e. weighted average of all the responses received. The RII method is one of the most utilized and having a good reliable value while ranking the attributes/factors using structured questionnaire survey

Relative importance index= $(4n_4+3n_3+2n_2+1n_1)/(A*N)$

n_4= number of healthcare professionals for very important

n_3 =number of healthcare professionals for important

n_2= number of healthcare professionals for less important

n_1=number of healthcare professionals for not important

weights for each scales

Very important = 4

important=3

less important=2

not important=1

A is the highest weight=4 (derived from very important)

N is the Total number of respondents = 582

Therefore

Relative importance index= $(4n_4+3n_3+2n_2+1n_1)/(A*N)$

Then rank the RII values range from 0 to 1

	Not important	Less important	Importan t	Very important	**N**	Summation of weights of Likert	A*N	**RII**	Rank
Scale	n=1	n=2	n=3	n=4		$(4n_4+3n_3+2n_2+1n_1)$		$4n_4+3n_3+2n_2+1n_1)/A*N$	
2. Prescribing antibacterial drugs when not needed	31	33	123	395	582	2046	2328	0.878866	1
3. Poor adherence of patients to prescribed antibiotic courses	14	44	184	340	582	2014	2328	0.86512	2
1. Prescribing the wrong antibacterial drugs	29	67	167	319	582	1940	2328	0.833333	3
6. Empirical (without lab investigation) antibiotic prescribing	25	76	167	314	582	1934	2328	0.830756	4
7. Poor access to antibiograms to guide prescription	30	79	219	254	582	1861	2328	0.799399	5
9. Lack of sufficient diagnostic laboratory facilities	44	83	174	281	582	1856	2328	0.797251	6
5. Lack of continuing education and updated information on antibiotics for pr...	39	74	216	253	582	1847	2328	0.793385	7
11. Lack of restriction controls on antibacterials access and prescription	40	83	225	234	582	1817	2328	0.780498	8
8. Use of antibacterials for longer duration than standard duration	49	94	184	255	582	1809	2328	0.777062	9
13. Poor quality antibacterials	72	126	171	213	582	1689	2328	0.725515	10
10. Lack/inadequate infection control in the health facility	60	124	228	170	582	1672	2328	0.718213	11
4. Poor access to treatment guidelines within hospital	67	139	184	192	582	1665	2328	0.715206	12

14. Pharmaceutical company influence	92	121	173	196	582	1637	2328	0.703179	13
12. Lack/shortage of antibacterials	87	130	205	160	582	1602	2328	0.688144	14

Prescribing antibacterial drugs when not needed **has a relative importance index (RII =**0.878866) and is ranked 1. This means healthcare professionals in the study gave more importance to prescribing antibacterials drugs when not needed as an important cause of antibacterial resistance

Table S4. relative importance ranking on possible causes of ABR (N=582).

	**Healthcare professionals in selected hospitals (N=582)**										
	**Nurses**	**PT**	**CO**	**MO**	**P**	**MS**	**LT**	**Total**	**RII**	**Rank**	**P Value**
	(n=199)	(n=30)	(n=136)	(n=121)	(n=24)	(n=50)	(n=22)	582			
Prescribing the wrong antibacterial drugs	162 (81.4)	23 (76.7)	114 (83.8)	99 (81.8)	23 (95.8)	44 (88)	21 (95.5)	486 (83.5)	0.833	3	0.69
Poor adherence of patients to prescribed antibacterial courses	176 (88.4)	28 (93.3)	123 (90.4)	107 (88.4)	24 (100.0)	49 (98.0)	17 (77.3)	524 (90.0)	0.865	2	0.13

Prescribing an antibacterial when not needed	177 (88.9)	27 (90.0)	119 (87.5)	104 (86.0)	23 (95.8)	50 (100.0)	18 (81.8)	518 (89.0)	0.879	1	0.15
Poor access to treatment guidelines within hospital	133 (66.8)	15 (50)	83 (61.0)	80 (66.1)	12 (50)	38 (76)	15 (68.2)	376 (64.6)	0.715	12	0.26
Empirical (without lab investigation) antibacterial prescribing	164 (82.4)	25 (83.3)	105 (77.2)	103 (85.1)	22 (91.8)	42 (84.0)	20 (90.9)	481 (82.6)	0.831	4	0.28
Poor access to antibiograms to guide prescription	156 (78.4)	23 (76.7)	106 (77.9)	99 (81.8)	21 (87.5)	49 (98.0)	19 (86.4)	473 (81.3)	0.799	5	*0.01
Lack of continuing education and updated information on antibacterials	161 (80.9)	24 (80.0)	101 (74.3)	97 (80.2)	18 (75.0)	49 (98)	19 (86.4)	469 (80.6)	0.793	7	0.52
Use of antibacterials for longer duration than standard duration	170 (85.4)	21 (70.0)	96 (70.0)	85 (70.2)	11 (45.8)	38 (76.0)	18 (81.8)	439 (75.4)	0.777	9	0.001
Lack of restriction controls on antibacterials	155 (77.9)	22 (73.3)	104 (76.5)	92 (76.0)	22 (91.7)	48 (96.0)	16 (72.7)	459 (78.9)	0.78	8	*0.04

access and prescription											
Lack of sufficient diagnostic laboratory facilities	151 (75.9)	26 (86.7)	104 (76.5)	96 (79.3)	19 (79.2)	43 (86.0)	16 (72.7)	455 (78.2)	0.797	6	0.91
Lack/inadequate infection control in the health facility	137 (68.8)	20 (66.7)	87 (64.0)	80 (66.1)	14 (58.3)	40 (80.0)	20 (90.9)	398 (68.4)	0.718	11	0.08
Lack/shortage of antibacterials	132 (66.3)	21 (70.0)	87 (64.0)	70 (57.9)	12 (50.0)	35 (70.0)	8 (36.4)	365 (62.7)	0.688	14	0.05
Poor quality antibacterials	122 (61.3)	20 (66.7)	98 (72.1)	76 (62.8)	12 (50.0)	43 (86.0)	13 (59.1)	384 (66.0)	0.726	10	0.11
Pharmaceutical company influence	121 (60.8)	21 (70)	94 (69.1)	74 (61.2)	13 (54.2)	37 (74.0)	9 (40.9)	364 (63.4)	0.703	13	0.28

PT: Pharmacy technician, CO: clinical officer, MO: medical officer, P: Pharmacist, LT: Laboratory technician, RII: Relative Importance Index

*Kruskal–Wallis test.

‡Rated on a Likert scale from (1= not important) to 4 (very important) and *shows significant difference at $p < 0.05$.

Table S5. Perspectives on adopted policies to support establishment of ASP in selected hospitals (N=582)

	Healthcare providers in selected hospitals (N=582)								
	**Nurses**	**PT**	**CO**	**MO**	**P**	**MS**	**LT**	**Total**	P value

	n=199 n(%)	n=30	n=136	n=121	n=24	n=24	n=22	N=582	
**Adopted hospital policies to support ASP development**									
Strengthened regulations on the distribution of high-quality antibacterials	144 (72.4)	24 (80)	99 (72.8)	86 (71.1)	18 (75)	33 (66)	15 (68.2)	**419 (72)**	0.899
Development and disseminating standard treatment guidelines	154 (77.4)	24 (80)	102 (75)	81 (66.9)	16 (66.7)	35 (70)	16 (72.7)	**428 (73.5)**	0.432
Participating in a nationwide or regional antibacterial awareness campaign	65 (32.7)	11 (36.7)	38 (27.9)	46 (38)	10 (41.7)	22 (44)	6 (27.3)	**198 (34)**	0.355
Regularly reviewing antibacterials from the national essential medicines lists (EML)	107 (53.8)	17 (56.7)	61 (44.9)	43 (35.5)	7 (29.2)	16 (32)	14 (63.6)	**265 (45.5)**	0.002*
Translating of international and national action plans on antibacterial resistance to hospital action plans	57 (28.6)	12 (40)	32 (23.5)	22 (18.2)	2 (8.3)	15 (30)	6 (27.3)	**146 (25.1)**	0.053
Implementing a Medicine Therapeutic Committee (MTC) antibacterial use	54 (27.1)	8 (26.7)	24 (17.6)	23 (19)	1 (4.2)	9 (18)	10 (45.5)	**129 (22.2)**	0.008*
Monitoring antibacterial consumption and identifying areas for improvement	74 (37.2)	10 (33.3)	40 (29.4)	39 (32.2)	6 (25)	15 (30)	9 (40.9)	**193 (33.2)**	0.689
Generating reports on antibacterial resistance to guide the prescription	43 (21.6)	9 (30)	27 (17.4)	21 (17.4)	2 (8.3)	5 (10)	8 (36.4)	**115 (19.8)**	0.07
Developed a functioning antimicrobial resistance surveillance system	39 (19.6)	4 (13.3)	20 (14.7)	17 (14)	2 (8.3)	9 (18)	4 (18.2)	**95 (16.3)**	0.701

Strengthening infection prevention and control measures	138 (69.3)	18 (60)	72 (52.9)	63 (52.1)	8 (33.3)	21 (42)	11 (50)	**331 (56.9)**	<0.001*

P.T.: Pharmacy technician, CO: clinical officer, MO: medical officer, P: Pharmacist, LT: Laboratory technician. *show significant difference at P < 0.05.

PT: Pharmacy technician, CO: clinical officer, MO: medical officer, P: Pharmacist, LT: Laboratory technician

*show significant difference at P < 0.05.

Table S6. Factors associated with low perspective scores on adopted hospital policies to support the establishment of ASP(N=582).

	Low scores n(%)	Moderate scores n(%)	high scores n(%)	COR (95% CI)	AOR (95% CI)	P-value
	367(63.1%)	151(25.9%)	64 (11.0%)			
**Sex**						
Male	223(38.3)	76 (13.1)	34 (5.8)	1.0	1.0	
Female	144(24.7)	75(12.9)	30 (5.2)	1.65 (1.24-2.20)	1.73 (1.28-2.34)	***<0.001**
Age						
30–39	157(27.0)	68(11.7)	21(3.6)	1.0	1.0	
20–29	67(11.5)	20(3.7)	9(1.5)	0.71 (0.47-1.08)	0.71 (0.46-1.10)	0.122

40–49	107(18.4)	41(7.0)	23(4.0)	1.13 (0.80-1.61)	1.04 (0.73-1.49)	0.814
50+	36(6.2)	22(3.8)	11(1.9)	1.81 (1.16-2.84)	1.92 (1.22-3.01)	***0.004**
**Department**						
Medicine	71 (12.2)	18 (3.1)	9(1.5)	1.0	1.0	
Surgery	42 (7.2)	21(3.6)	8(1.4)	1.61 (0.98-2.66)	1.63 (0.95-2.80)	0.078
Paediatrics	73 (12.5)	25(4.3)	11(1.9)	1.10 (0.69-1.76)	1.11(0.68-1.82)	0.671
Pharmacy	33 (5.7)	10(1.7)	7(1.2)	1.25 (0.70-2.24)	1.71 (0.91-3.22)	0.096
Obstetrics and Gynaecology	49 (8.4)	32(5.5)	12(2.1)	1.88 (1.15-3.08)	1.73(1.03-2.90	***0.037**
Outpatient	67 (11.5)	24(4.1)	10(1.7)	1.16 (0.71-1.87)	1.10 (0.67-1.81)	0.71
Others	32(5.5)	21(3.6)	7(1.2)	1.53 (0.85-2.75)	1.77 (0.97-3.24)	0.064
**Region**						
Central	136(23.4)	42(7.2)	15(2.6)	1.0	1.0	
North	34(5.8)	16(2.7)	17(2.9)	2.57 (1.43-4.61)	2.97(1.63-5.42)	***<0.001**
East	100(17.2)	63(10.8)	11(1.9)	1.54 (1.10-2.15)	1.47 (1.03-2.09)	***0.034**
West	97(16.7)	30(5.2)	21(3.6)	1.24 (0.85-1.82)	1.28 (0.87-1.88)	0.214
