# Supplementary material for: Antimicrobial Stewardship Programmes: Healthcare Providers’ Perspectives on Adopted Hospital Policies That Combat Antibacterial Resistance in Selected Health Facilities in Uganda
Source: Antibiotics (Basel). 2024 Oct 23;13(11):999. doi: 10.3390/antibiotics13110999 (PMC11590866; doi:10.3390/antibiotics13110999)
Supplement: Supplementary file 1 [file antibiotics-13-00999-s001.zip › File S4. Ethical clearance.pdf]

Adopted hospital policy actions to combat antibacterial resistance

Ethical clearance letters

MAKERERE UNIVERSITY

P.O. Box 7072 Kampala, Uganda
E-mail: biomedicalresearch62@gmail.com

Phone:256752575050
Fax: 256 414 532204

COLLEGE OF HEALTH SCIENCES
SCHOOL OF BIOMEDICAL SCIENCES
HIGHER DEGREES RESEARCH AND ETHICS COMMITTEE

23rd Jan 2019

SBS-624

To Mr. Kimbowa Isaac
Principal Investigator
Department of Pharmacology

Category of review
[X] Initial review
[ ] Continuing review
[ ] Amendment
[ ] Termination of study
[ ] SAEs

Decision of the School of Biomedical Sciences Higher Degrees Research and Ethics Committee (SBS-HDREC) at its 88th REC meeting held on 13th Dec 2018.

In the matter concerning the review of a PhD proposal entitled, **"Antimicrobial stewardship practices and quality of antibacterial use in children under 5 years in selected health facilities in Uganda." SBS-HDREC - 624**

The investigators have met all the requirements as stated by **SBS-HDREC** and therefore, the proposal is **APPROVED.**

The approval granted includes all materials submitted by the investigators for **SBS-HDREC** review including;

1. Protocol
2. Study tools
3. Consent forms

and is valid until **12th Dec 2019**.

Please note that the annual report and the request for renewal where applicable, should be submitted six weeks before expiry date of approval.

Any problems of a serious nature related to the execution of the research protocol should be promptly reported to the **SBS-HDREC**, and any changes to the research protocol should not be implemented without approval from **SBS-HDREC**, except when necessary to eliminate apparent immediate hazards to the research participant(s)

You are required to register the research protocol with the Uganda National Council for Science and Technology **(UNCST)** for final clearance to undertake the study in Uganda.

Signed............................

MAKERERE UNIVERSITY
SCHOOL OF BIOMEDICAL SCIENCES
VALID UNTIL
12 DEC 2019
RESEARCH & ETHICS COMMITTEE
P.O. BOX 7072, KAMPALA

Dr. Erisa Mwaka
Chairperson, School of Biomedical Sciences Higher Degrees Research and Ethics Committee.
