# Supplementary material for: Antimicrobial Stewardship Programmes: Healthcare Providers’ Perspectives on Adopted Hospital Policies That Combat Antibacterial Resistance in Selected Health Facilities in Uganda
Source: Antibiotics (Basel). 2024 Oct 23;13(11):999. doi: 10.3390/antibiotics13110999 (PMC11590866; doi:10.3390/antibiotics13110999)
Supplement: Supplementary file 1 [file antibiotics-13-00999-s001.zip › File S5. ethical clearance.pdf]

Uganda National Council for Science and Technology

(Established by Act of Parliament of the Republic of Uganda)

Dear Isaac Kimbowa ,

I am pleased to inform you that on **24/04/2019**, the Uganda National Council for Science and Technology (UNCST) approved the above referenced research project. The Approval of the research project is for the period of **24/04/2019** to **24/04/2021**.

Your research registration number with the UNCST is **HS339ES**. Please, cite this number in all your future correspondences with UNCST in respect of the above research project. As the Principal Investigator of the research project, you are responsible for fulfilling the following requirements of approval:

1. Keeping all co-investigators informed of the status of the research.

2. Submitting all changes, amendments, and addenda to the research protocol or the consent form (where applicable) to the designated Research Ethics Committee (REC) or Lead Agency for re-review and approval **prior** to the activation of the changes. UNCST must be notified of the approved changes within five working days.

3. For clinical trials, all serious adverse events must be reported promptly to the designated local REC for review with copies to the National Drug Authority and a notification to the UNCST.

4. Unanticipated problems involving risks to research participants or other must be reported promptly to the UNCST. New information that becomes available which could change the risk/benefit ratio must be submitted promptly for UNCST notification after review by the REC.

5. Only approved study procedures are to be implemented. The UNCST may conduct impromptu audits of all study records.

6. An annual progress report and approval letter of continuation from the REC must be submitted electronically to UNCST. Failure to do so may result in termination of the research project.

Please note that this approval includes all study related tools submitted as part of the application as shown below:

No.	Document Title	Language	Version Number	Version Date

1	questionnaire and interview guide sub-study I	English	APPROVED	01 February 2019
2	questionnaire sub-study II	English	APPROVED	01 February 2019
3	Medical record data extract guide and key informant interview guide for sub-study IV	English	APPROVED	01 February 2019
4	Medical record data extract guide and key informant interview guide for sub-study IV	English	APPROVED	01 February 2019
5	informed consent for questionnire and key informant study for sub-study I	English	APPROVED	01 February 2019
6	informed consent for questionnaire for sub-study II	English	APPROVED	01 February 2019
7	informed consent for key informant interview for sub-study III	English	APPROVED	01 February 2019
8	informed consent for key informed interview for sub-study IV	English	APPROVED	01 February 2019
9	consent waiver for medical record review for sub-study III and IV	English	APPROVED	01 February 2019

Yours sincerely,

Hellen Opolot
For: Executive Secretary
UGANDA NATIONAL COUNCIL FOR SCIENCE AND TECHNOLOGY
